# Supplementary material for: Synergistic Mn-Co catalyst outperforms Pt on high-rate oxygen reduction for alkaline polymer electrolyte fuel cells
Source: Nat Commun. 2019 Apr 3;10:1506. doi: 10.1038/s41467-019-09503-4 (PMC6447550; doi:10.1038/s41467-019-09503-4)
Supplement: Supplementary file 1 — Supplementary Information [file 41467_2019_9503_MOESM1_ESM.pdf]

Supplementary Information

Synergistic Mn-Co Catalyst Outperforms Pt on High-Rate Oxygen Reduction for Alkaline Polymer Electrolyte Fuel Cells

Ying Wang[1,2], Yao Yang[3], Shuangfeng Jia[4], Xiaoming Wang[5], Kangjie Lyu[1], Yanqiu Peng[1], He Zheng[4], Xing Wei[1], Huan Ren[1], Li Xiao[1,*], Jianbo Wang[2,4], David A. Muller[6], Héctor D. Abruña[3,*], Bing Joe Hwang[5], Juntao Lu[1], and Lin Zhuang[1,2,*]

[1] *College of Chemistry and Molecular Sciences, Hubei Key Lab of Electrochemical Power Sources, Wuhan University, Wuhan 430072, China*

[2] *The Institute for Advanced Studies, Wuhan University, Wuhan 430072, China*

[3] *Department of Chemistry and Chemical Biology, Baker Lab, Cornell University, Ithaca, New York 14853, USA*

[4] *School of Physics and Technology, Center for Electron Microscopy, MOE Key Laboratory of Artificial Studies, Wuhan University, Wuhan 430072, China*

[5] *Department of Chemical Engineering, National Taiwan University of Science and Technology, Taipei 10607, Taiwan*

[6] *School of Applied and Engineering Physics, Cornell University, Ithaca, New York 14853, USA*

* Corresponding authors.

E-mails: chem.lily@whu.edu.cn, hda1@cornell.edu, lzhuang@whu.edu.cn

Supplementary Figures and Tables

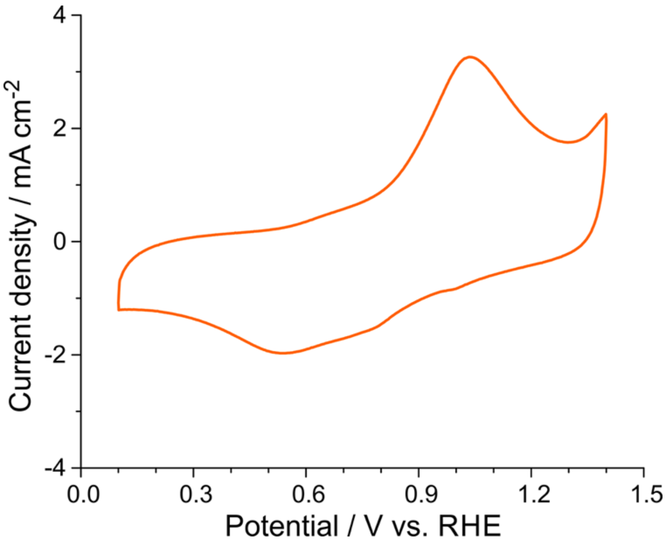

Supplementary Figure 1 Cyclic voltammogram at 50mV/s of the MCS catalyst in deoxygenated 1.0 M KOH solution.

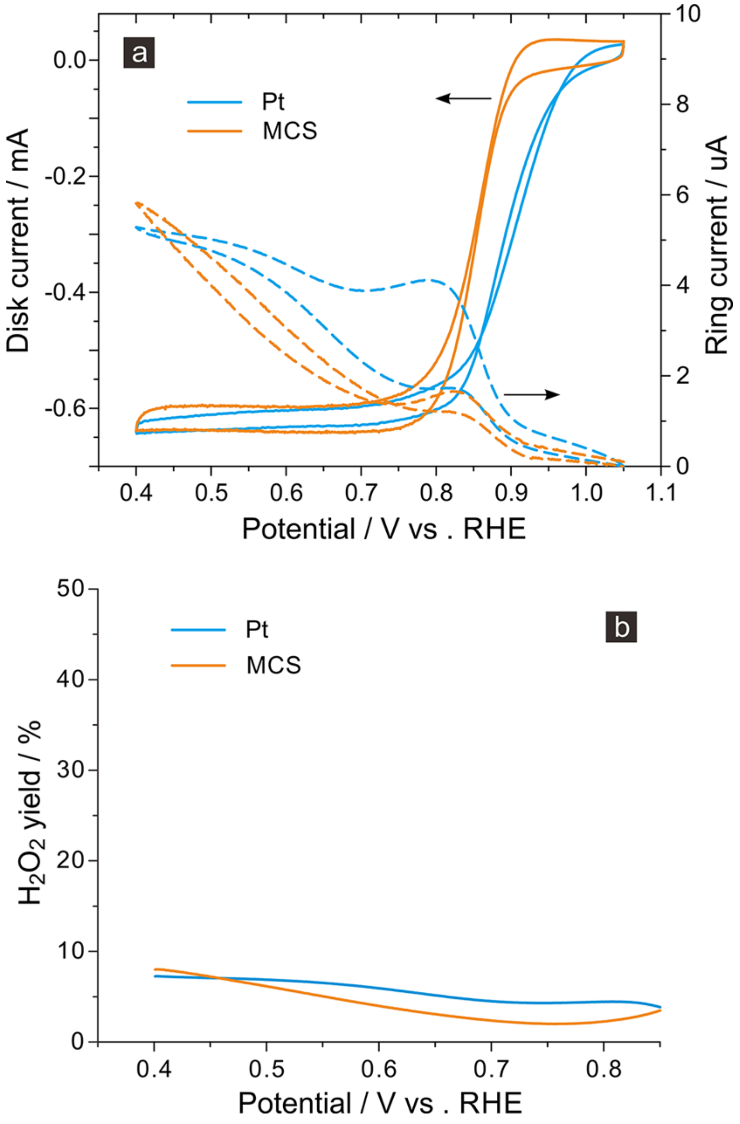

Supplementary Figure 2 RRDE results. (a) Disk current and ring current recorded in O_2-saturated 1.0 M KOH solution. (b) The calculated H_2O_2 yield percentage (%). Sweep rate: 5 mV s^{-1}.

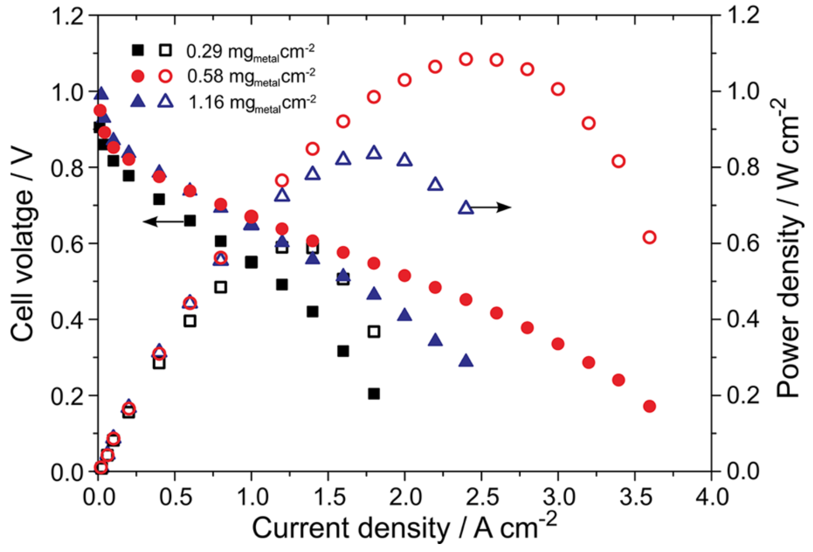

Supplementary Figure 3 APEFC cell performances using different loadings of MCS catalyst. Single Cells were operated at 60°C. Anode catalyst: 60 wt% Pt-Ru/C (Johnson Matthey, 0.4 mg_{metal} cm^{-2}). Alkaline polymer electrolyte: *a*QAPS-S_8 membrane (35 μm in thickness) and *a*QAPS-S_{14} ionomer (20 wt% in electrode) (*5*). 100% RH humidified H_2 and O_2 were fed at a flow rate of 200 mL min^{-1} with a backpressure of 0.1 MPa.

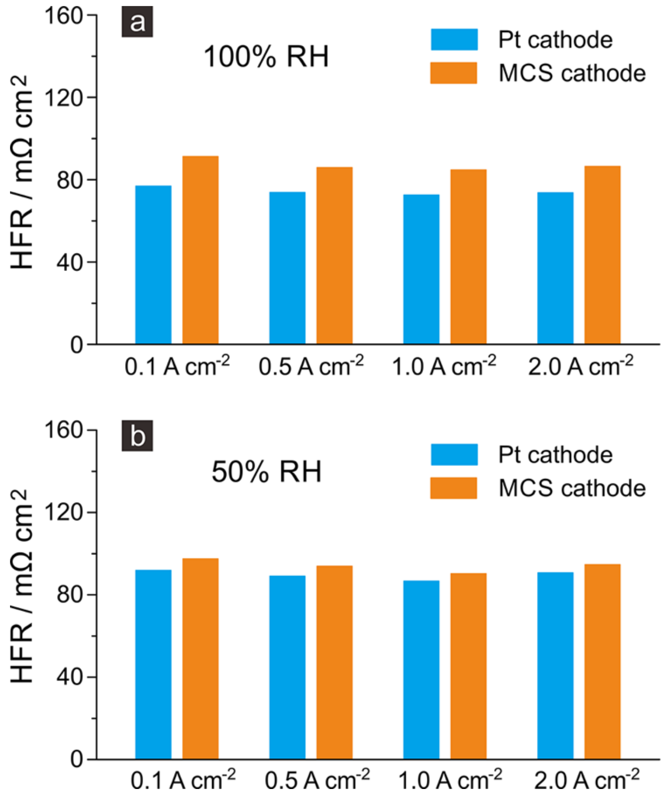

Supplementary Figure 4 High-frequency resistance (HFR) of the APEFC single cells operated at different current densities and under different RH of the reactant gases. (a) 100% RH; (b) 50% RH.

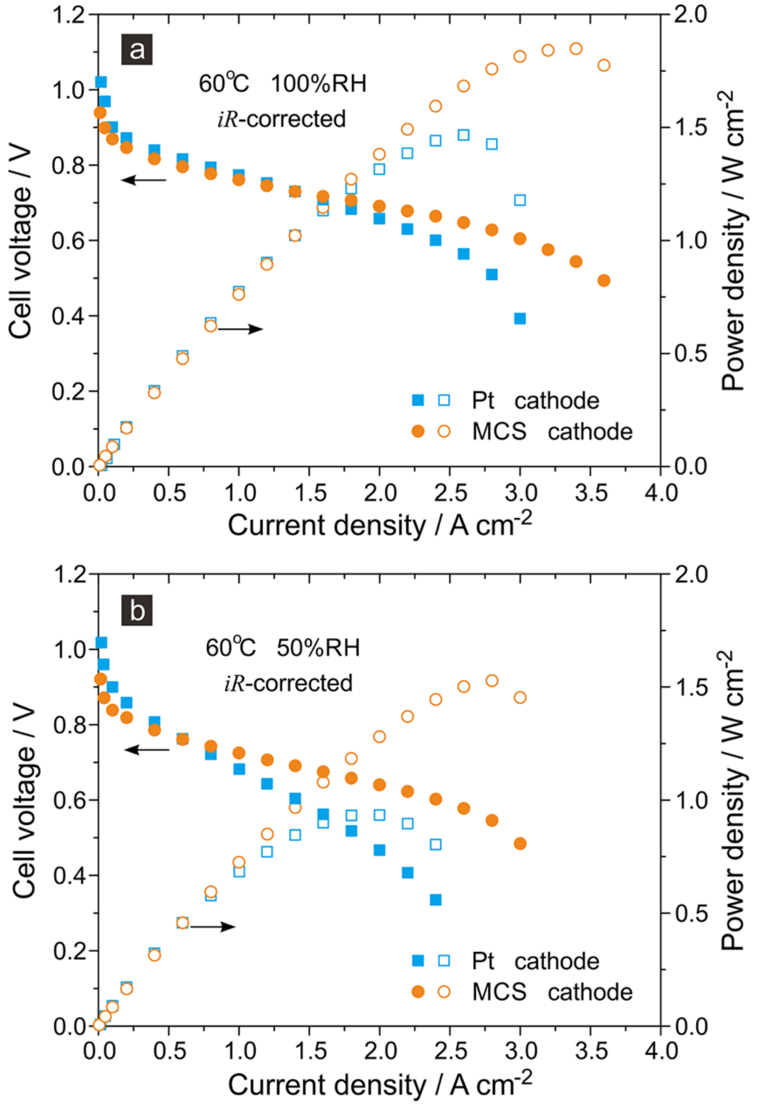

Supplementary Figure 5 *iR* corrected APEFC cell performance. (a) Under 100% RH; (b) under 50% RH.

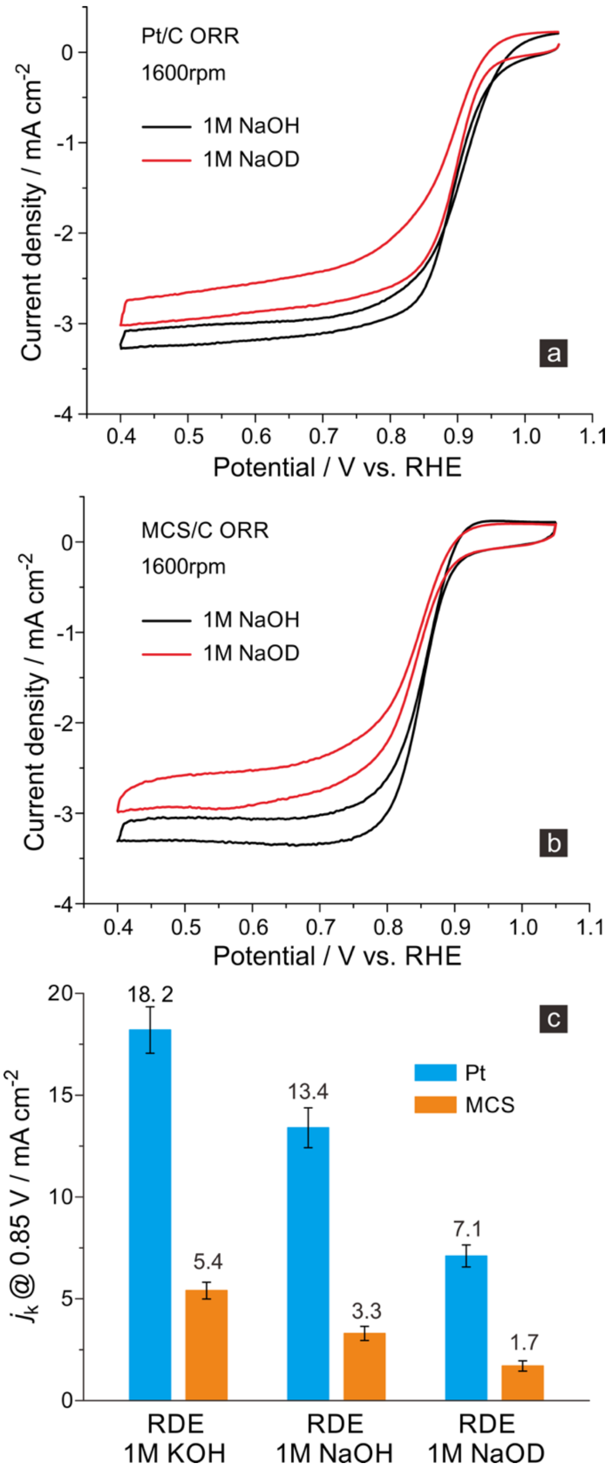

Supplementary Figure 6 Results from isotopic labelling experiments. (a & b) RDE tests for Pt and MCS catalysts, respectively, in O_2-saturated alkaline solutions. Scan rate = 5 mV s^{-1}. (c) Comparison of kinetic current densities (j_k) at 0.85 V (vs. RHE).

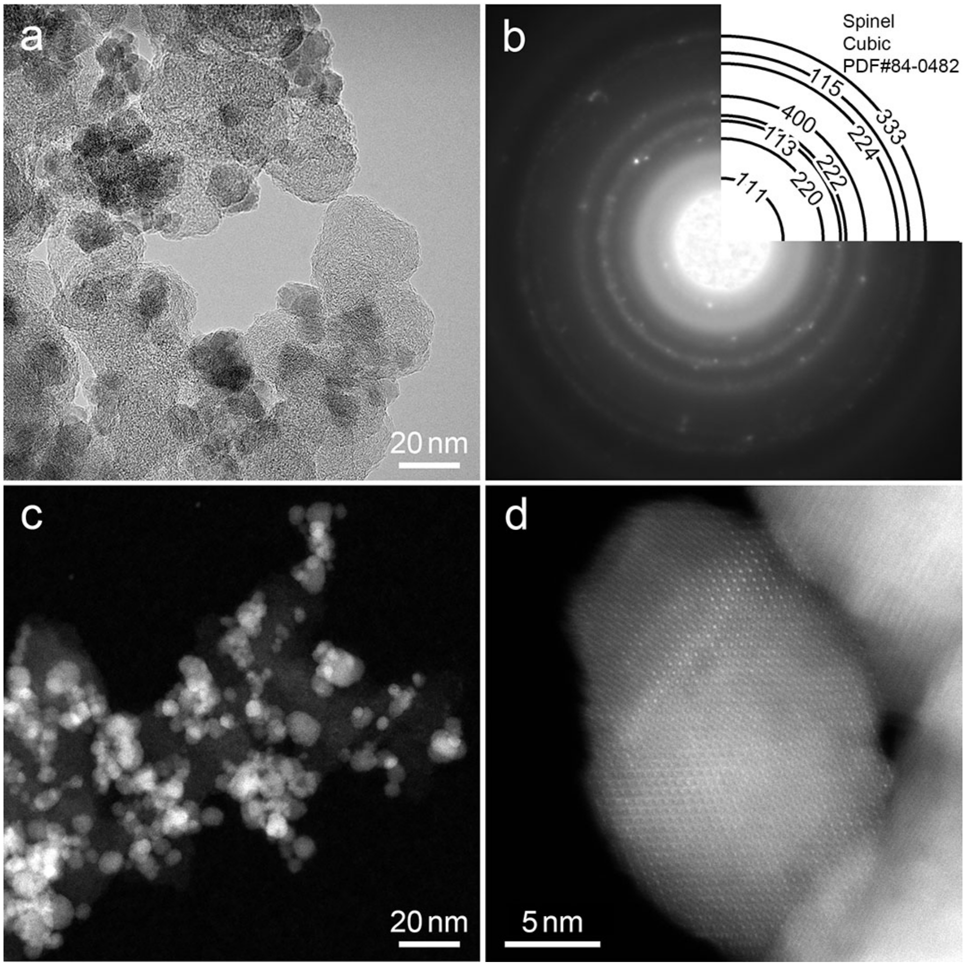

Supplementary Figure 7 STEM observation of MCS/C. (a) Bright-field image of MCS/C. (b) Electron diffraction pattern. (c) Dark-field image of MCS/C. (d) High-resolution dark-field image of MCS particles.

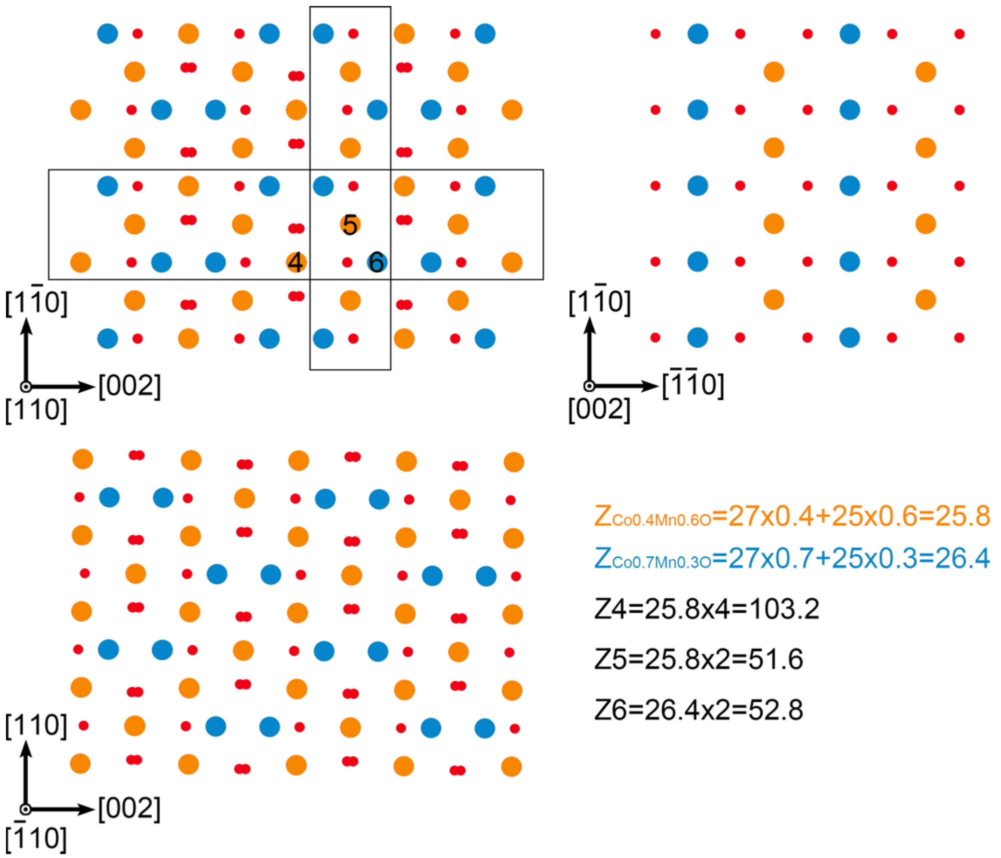

Supplementary Figure 8 Interpretation of the brightness of the HAADF-STEM image taken along [110] zone axis (Fig. 2c).

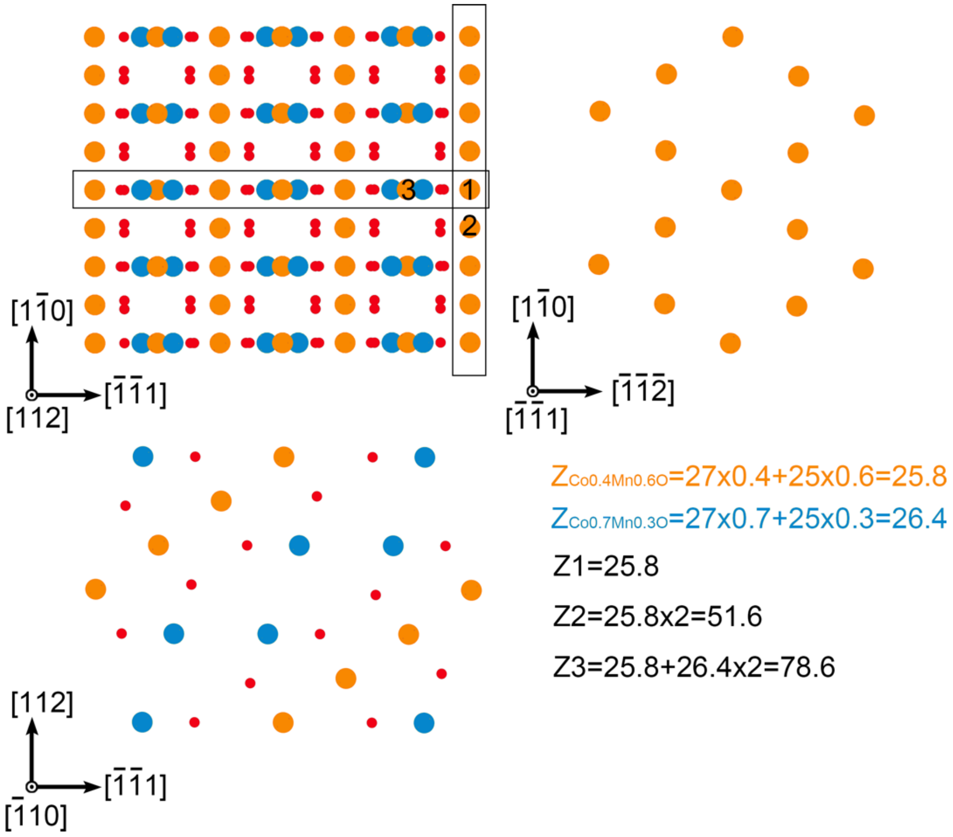

Supplementary Figure 9 Interpretation of the brightness of the HAADF-STEM image taken along [112] zone axis (Fig. 2d).

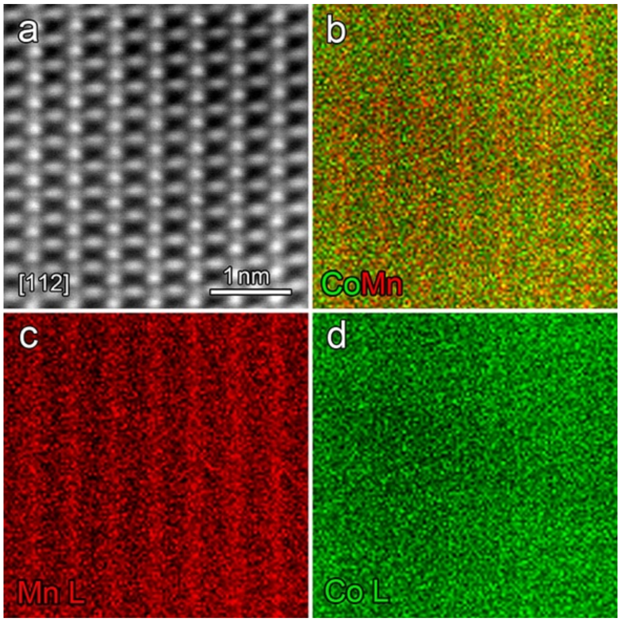

Supplementary Figure 10 STEM-EDX mapping confirms the enrichment of Mn at the B site of the spinel lattice. (a) HAADF-STEM image taken along [112] zone axis. (b) Addition of the Co and Mn signals. (c) Mn L-edge signal (d) Co L-edge signal.

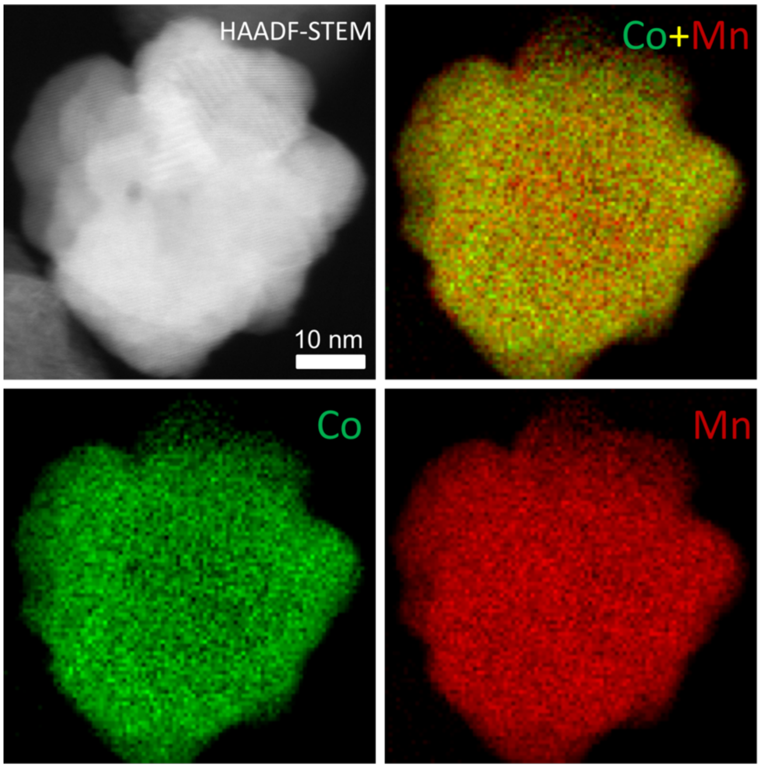

Supplementary Figure 11 STEM-EELS mappings of a MCS particle.

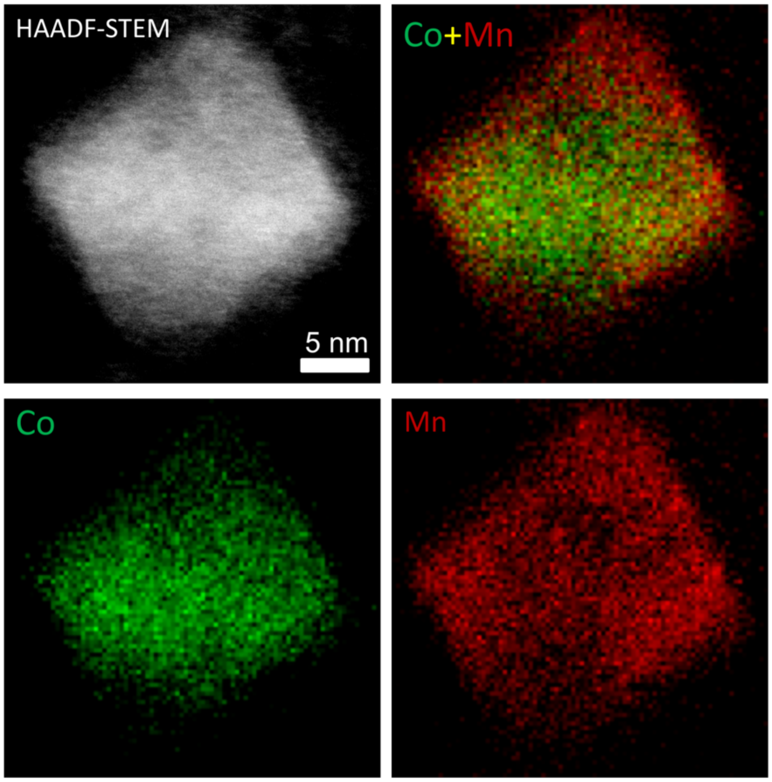

Supplementary Figure 12 STEM-EELS mappings of an Mn-MCS particle, showing the segregation of Mn at the particle surface.

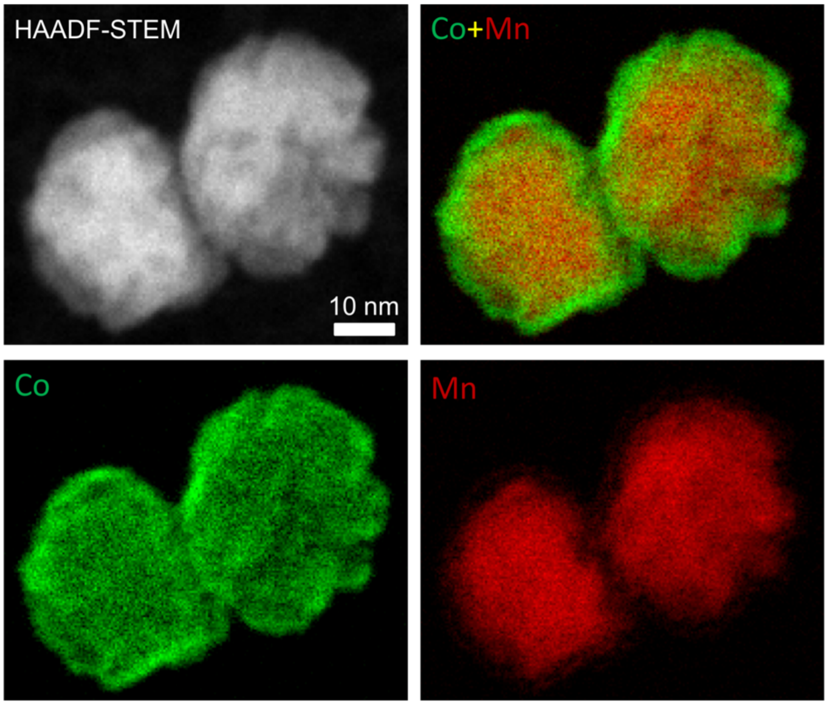

Supplementary Figure 13 STEM-EELS mappings of Co-MCS particles, showing the segregation of Co at the particle surface.

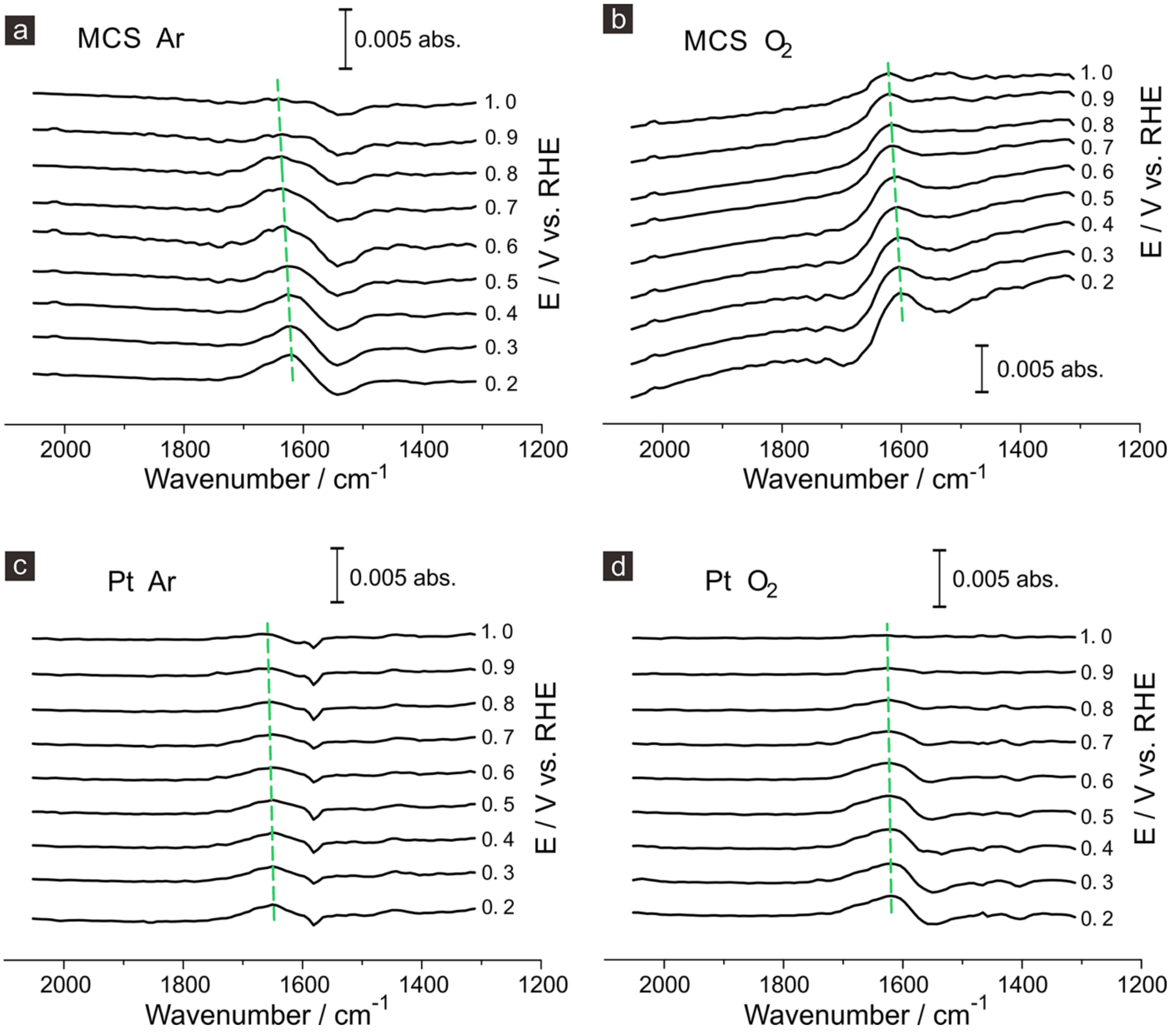

Supplementary Figure 14 *In-situ* ATR-FTIR spectra recorded in 1.0 M KOH solution under different potentials. The reference potential is 1.2 V (*vs.* RHE). (a) MCS/C catalyst under Ar atmosphere. (b) MCS/C catalyst under O_2 atmosphere. (c) Pt/C catalyst under Ar atmosphere. (d) Pt/C catalyst under O_2 atmosphere.

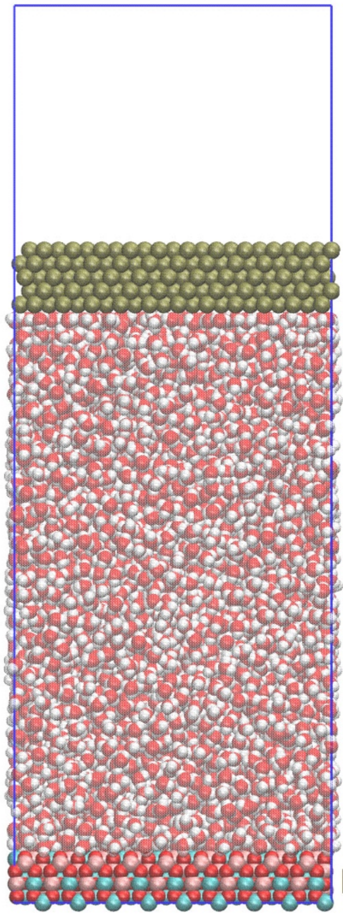

Supplementary Figure 15 Model for MD simulation of water on MCS (100) and Pt (111) surfaces.

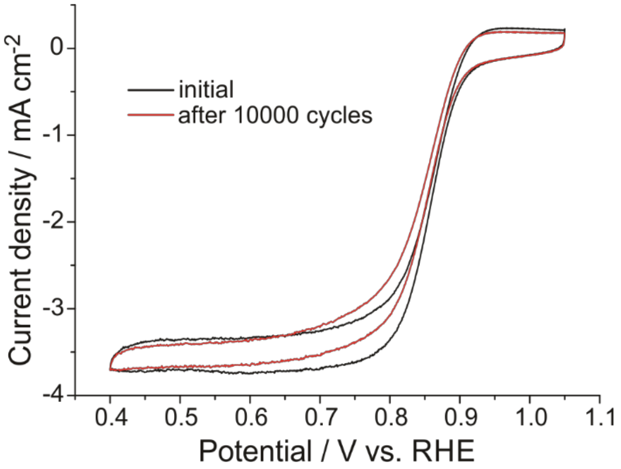

Supplementary Figure 16 Stability test of MCS in O_2-saturated 1.0 M KOH solution. The stability was evaluated under 10000 potential cycles from 0.6 V to 1.0 V at 0.1 V s^{-1}. The rotation rate of RDE test was 1600 rpm, and the scan rate was 5 mV s^{-1}.

Supplementary Table 1 DFT-calculated adsorption energies of oxygen molecule (O_2) on MCS(100) surface.

Adsorption site**	Stable structure	$\Delta E_{ads}(O_2)^*$
Mn site<br>MCS(100)	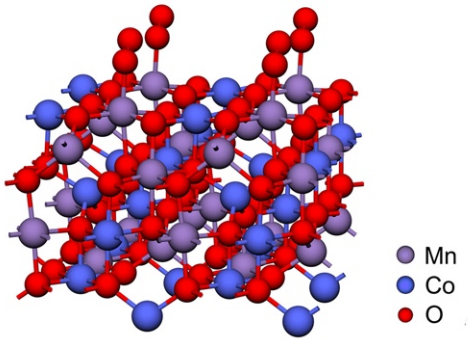	–1.58 eV
Mn site<br>MCS(100)	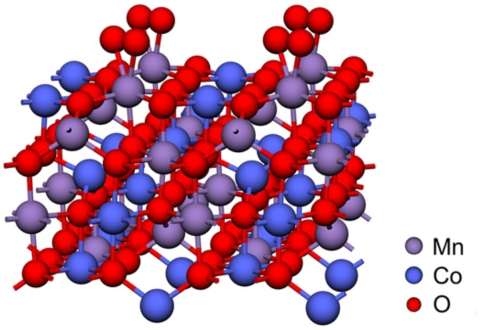	–1.48 eV
Mn-Co bridge-site<br>MCS(100)	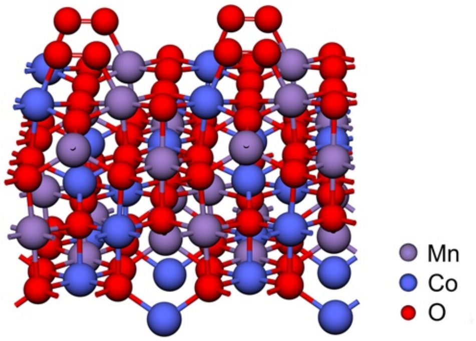	–1.64 eV

* The adsorption energy of O_2 is defined as $\Delta E_{ads}(O_2) = E(M\text{-}O_2) - E(M) - E(O_2)$.

** No stable structure was found for O_2 adsorption alone on the Co site.

Supplementary Table 2 DFT-calculated adsorption energies of oxygen molecule (O_2) on Pt(111) surface.

Adsorption site	Stable structure	$\Delta E_{ads}(O_2)^*$
t-b-t<br>Pt(111)	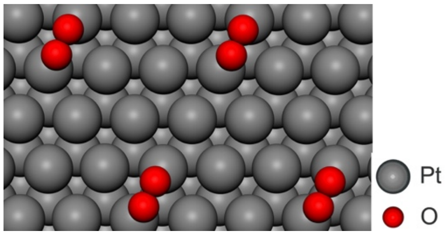	–1.83 eV
t-f-b<br>Pt(111)	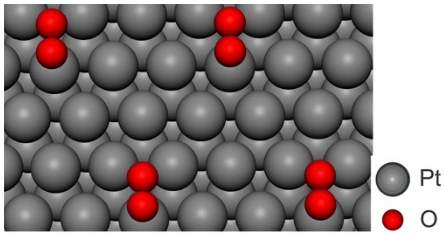	–1.65 eV
t-h-b<br>Pt(111)	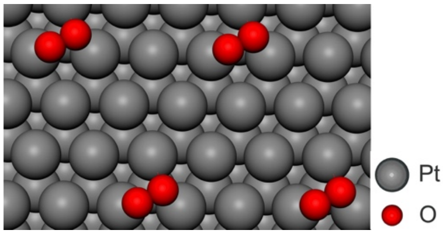	–1.58 eV
fcc<br>Pt(111)	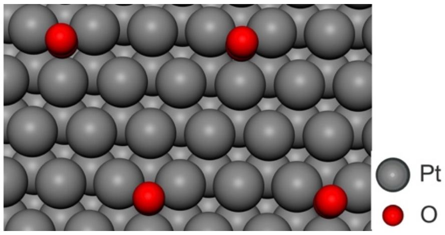	–0.85 eV

* The adsorption energy of O_2 is defined as $\Delta E_{ads}(O_2) = E(M\text{-}O_2) - E(M) - E(O_2)$.

Supplementary Table 3 DFT-calculated adsorption energies of atomic oxygen (O) on MCS(100) surface.

Adsorption site	Stable structure	$\Delta E_{ads}(O)^*$
Mn site<br>MCS(100)	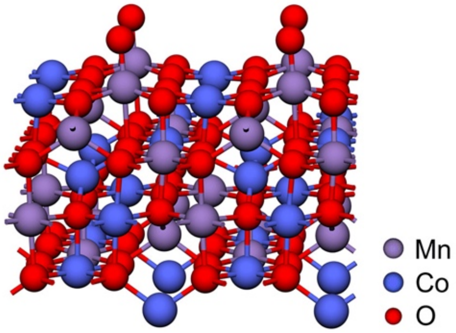	–1.48 eV
Co site<br>MCS(100)	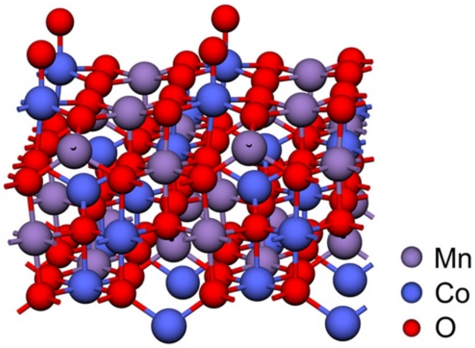	0.17 eV

* The adsorption energy of O is defined as $\Delta E_{ads}(O) = E(M\text{-}O) – E(M) – 1/2E(O_2)$, such that negative values of $\Delta E_{ads}(O)$ imply spontaneous dissociation of O_2 on the studied site.

Supplementary Table 4 DFT-calculated adsorption energies of atomic oxygen (O) on Pt(111) surface.

Adsorption site	Stable structure	$\Delta E_{ads}(O)^*$
fcc<br>Pt(111)	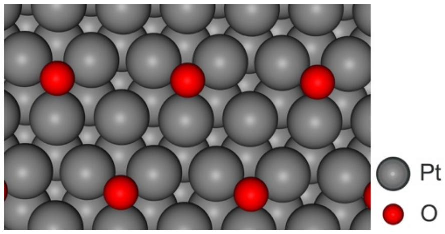	–1.12 eV
hcp<br>Pt(111)	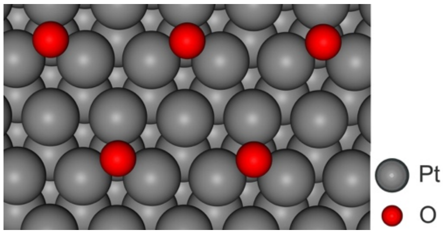	–0.76 eV
bridge<br>Pt(111)	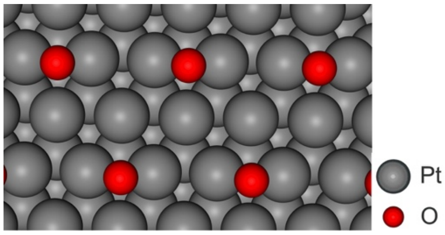	–0.58 eV
atop<br>Pt(111)	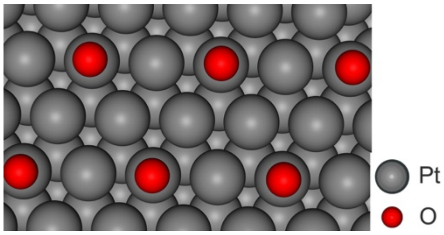	0.41 eV

* The adsorption energy of O is defined as $\Delta E_{ads}(O) = E(M\text{-}O) - E(M) - 1/2E(O_2)$, such that negative values of $\Delta E_{ads}(O)$ imply spontaneous dissociation of O_2 on the studied site.

Supplementary Table 5 DFT-calculated adsorption energies of water molecule (H_2O) on MCS(100) surface.

Adsorption site	Stable structure	$\Delta E_{ads}(H_2O)^*$
Mn site<br>MCS(100)	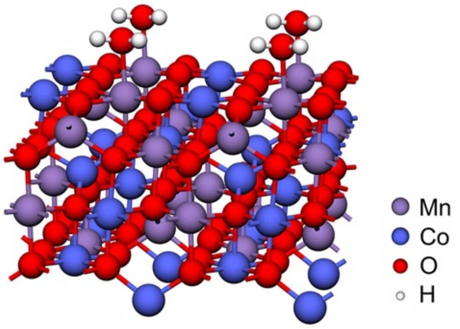 	–0.67 eV
Co site<br>MCS(100)	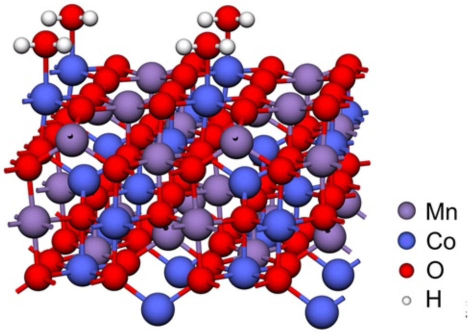 	–0.65 eV

* The adsorption energy of H_2O is defined as $\Delta E_{ads}(H_2O) = E(\text{M-H}_2\text{O}) - E(\text{M}) - E(\text{H}_2\text{O})$.

Supplementary Table 6 DFT-calculated adsorption energies of water molecule (H_2O) on Pt(111) surface.

Adsorption site	Stable structure	$\Delta E_{ads}(H_2O)^*$
atop<br>Pt(111)	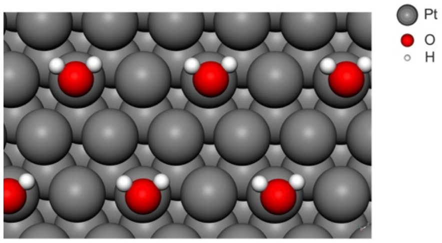	–0.22 eV
hcp<br>Pt(111)	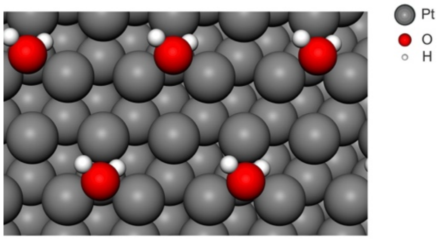	–0.11 eV
fcc<br>Pt(111)	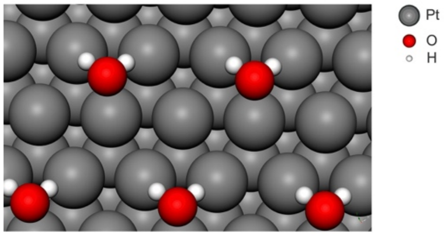	–0.09 eV
bridge<br>Pt(111)	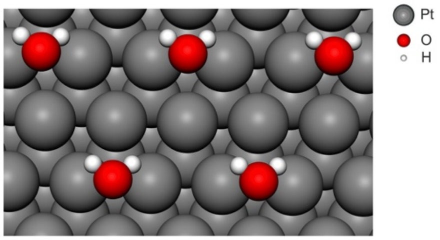	–0.09 eV

* The adsorption energy of H_2O is defined as $\Delta E_{ads}(H_2O) = E(M\text{-}H_2O) - E(M) - E(H_2O)$.

Supplementary Table 7 DFT-calculated energy barrier for Reaction I* on MCS(100).

Reaction coordinate	Structure	Energy change
Initial State	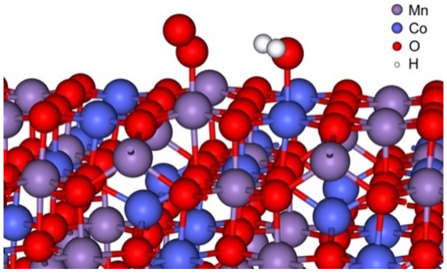	0 eV
Transition State	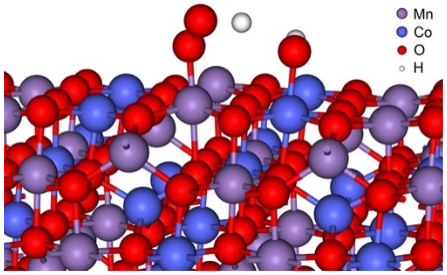	0.47 eV
Final State	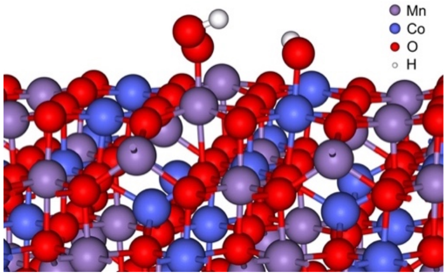	0.35 eV

* Reaction I: Mn-O_2 + Co-OH_2 → Mn-O_2H + Co-OH

Supplementary Table 8 DFT-calculated energy barrier for Reaction II* on MCS(100).

Reaction coordinate	Structure	Energy change
Initial State	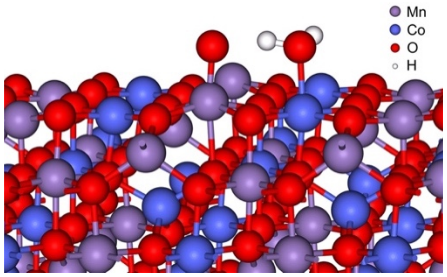	0 eV
Transition State	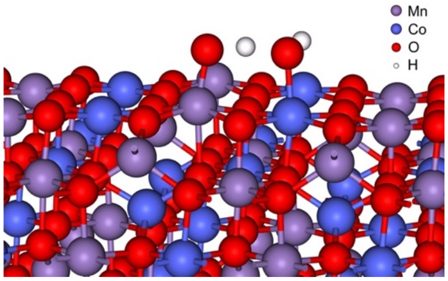	0.16 eV
Final State	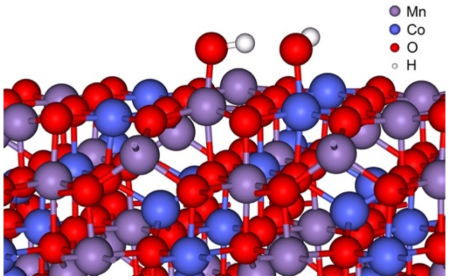	0.14 eV

* Reaction II: Mn-O + Co-OH_2 → Mn-OH + Co-OH
